# Chemical and spectroscopic signatures of resins from Sumatra (Sarolangun mine, Jambi Province) and Germany (Bitterfeld, Saxony-Anhalt)

**DOI:** 10.1038/s41598-020-74671-z

**Published:** 2020-10-26

**Authors:** Przemysław Drzewicz, Beata Naglik, Lucyna Natkaniec-Nowak, Magdalena Dumańska-Słowik, Paweł Stach, Mirosław Kwaśny, Jakub Matusik, Rastislav Milovský, Janusz Skonieczny, Dorota Kubica-Bąk

**Affiliations:** 1 Polish Geological Institute-National Research Institute, Rakowiecka 4, 00-975 Warszawa, Poland; 2 Polish Geological Institute-National Research Institute, Upper Silesian Branch, Królowej Jadwigi 1, 41-200 Sosnowiec, Poland; 3 AGH UST, University of Science and Technology, Faculty of Geology, Geophysics and Environmental Protection, Mickiewicza 30, 30-059 Kraków, Poland; 4 Institute of Optoelectronics, Military University of Technology, Gen. S. Kaliskiego 2, 00-908 Warszawa, Poland; 5 Earth Science Institute, Slovak Academy of Sciences, Ďumbierska 1, 974 11 Banská Bystrica, Slovakia; 6 Łukasiewicz Research Network-Polish Center for Technology Development, Stabłowicka 147, 54-066 Wrocław, Poland

**Keywords:** Solid Earth sciences, Mineralogy

## Abstract

Fossil resins from Miocene coal deposit (Sarolangun mine, Jambi Province, Sumatra, Indonesia) have been analysed using spectroscopic methods: Raman Spectroscopy (RS), Fourier Transform-Infrared Spectroscopy (FT-IR), ^13^C Nuclear Magnetic Resonance (^13^C NMR), Fluorescence Spectroscopy (FS), and Gas Chromatography–Mass Spectrometry (GC–MS) in order to describe their diagnostic features. Simultaneously, glessite, a fossil resin from Upper Oligocene Bitterfeld deposit (Saxony-Anhalt, Germany), originating from similar botanical sources (i.e. angiosperms) was tested with the same analytical methods in order to find similarities and differences between the resins. The resins differ in colour, transparency and amounts of inclusions (resins from Sumatra—yellow, and transparent with few inclusions; glessite—brown–red, translucent with wealth of inclusions). In general, the IR and RS spectra of these resins are very similar, probably because the glessite colour-changing additives can be very subtle and non-observable in the infrared region. The RS spectra revealed also a slight difference in intensity ratio of the 1650/1450 cm^−1^ bands (0.56 and 0.68 for Sumatra and Germany resins, respectively), indicating a differences in their maturation process. The resins from Sumatra seem to be more mature than glessite from Germany. The excitation–emission (EM–EX) and synchronous spectra showed unique, chemical compositions of these resins, which are different one from another. The GC–MS data for Sumatran resins, dominated by sesquiterpenoids and triterpenoids (amyrin), confirmed their botanical origin (angiosperms as their biological affinities). The sesquiterpenoid biomarkers with cadine-structures suggested the glessite underwent more advanced polymerization processes, which does not correlate with its RS spectrum. The geological factors, the environmental conditions of resin deposition, and later various diagenesis processes may have influenced the maturation and crosslinking of compounds. Despite the genetic similarity of the resins from various part of the world, Sumatra and Germany, advanced techniques such as Gas Chromatography–Mass Spectrometry and Fluorescence Spectroscopy were the most useful to find the differences between them. These differences are predominantly a result of different diagenetic transformations of the resins.

## Introduction

The fossil resins are transformation products of exudates from coniferous and deciduous trees that existed on Earth from millions of years ago. They were preserved to present day mainly due to compound polymerization and cross-linking, taking place during their diagenetic or/and catagenetic alterations^1^. Fossil resins are found in sediments of geological age spanning from Carboniferous to Cenozoic; however, they are mostly accumulated in layers dating from the Paleogene and Neogene periods^2^. In many cases, it is believed that the age of the resins is the same as the age of the resin-bearing sediments; however such assessment may be inaccurate due to the frequent redeposition of resins in younger geological environments. The complex transportation and post-depositional processes affect the chemical composition and spectroscopic properties of resins.

The age and origin as well as the diagenetic alterations of fossil resin in various geological environments worldwide are still disputable despite numerous investigations^3,4^. It is believed that many of the fossil resin features such as microhardness^5–7^, spectroscopic fingerprints^8–10^ and thermal properties^11–13^ may correlate with their age. In earlier investigations, it was found that the maturation of fossil resins has been influenced by various geological factors (e.g. volcanism, hydrothermal heating), not aging itself^8,10,11,14–16^.

The widely accepted classification system of fossil resins, proposed by Anderson and Winans^17^, Anderson et al.^18^ and Anderson and Botto^19^, is only based on chemotaxonomy. Hence, fossil resins, occurring in various parts of the world, but showing similar chemical compositions that are frequently caused by close affinity with the parent trees, are classified in the same group, e.g., Indonesian resins are found in the same group as glessite from Germany. Sumatran resins are from the trees of the *Dipterocarp*aceae species, belonging to the angiosperms group^20–24^. Leelawatanasuk et al.^25^ found they could be easily distinguished from resins of other localities worldwide (Baltic areas, Dominican Republic and Myanmar) based on their distinct inclusions and characteristic IR spectrum. Recently, resins from the Sarolangun mine in Sumatra (Jambi Province) were studied by Naglik et al.^16^. Three varieties of Sumatran resins, that formed one large aggregate, differed in colour, transparency and amounts of impurities. The first resin type (I) had a light-yellow colour, a high transparency, and relatively small number of inclusions. The second (II) was represented by translucent resins of colour graduating from orange to brown. This variety was mainly accumulated outside the tree trunk, and hence, hosted a greater number of different natural, organic and mineral inclusions. The third variety (III) was a white–grey and opaque type of resin with a foam-like structure. It was also most likely to have been secreted outside the tree trunk, and then flowed down to the forest bedding. Contrary to the Indonesian resins, the late Oligocene glessites from Bitterfeld were extensively investigated by Krumbiegel, Kosmowska-Ceranowicz and Wolfe^26–33^.

Glessite is defined as being “opaque reddish brown to brown fossil resins with some angiosperms as its botanical source”^34^. Kosmowska-Ceranowicz and Vávra^35^ noted that the infra-red spectra of Sumatran resins were very similar to the shape of IR spectra of glessite found in the Bitterfeld deposit (Saxony-Anhalt, Germany). Recently, Matuszewska^9^ discussed the structural similarity of resins from Borneo to resins of glessite type from Bitterfeld. The similarity of the resins IR spectra was explained by similarity of chemical composition, primarily to the content of cadinene type compounds (sesquiterpene group), and triterpenoids in those resins. Matuszewska^9^ did not find sufficient proof to confirm that origins of both resins are trees belonging to the family of close kinship. As a result, the name glessite in relation to Malaysian resins was disputable.

The main aim of this study is to elucidate the source of the Sumatran resins and their transformation pathways through various geological processes. Hence, a comparison of fossil resins originating from similar botanical sources, but from different environments was made, in order to reveal the influence of the palaeogeographical provenience and geological processes affecting the chemical composition and spectroscopic characteristics of the analytical material. Accordingly, the study is focused on the fossil resins from Sumatra (Jambi Province, Sarolangun) and glessite from Germany (Saxony-Anhalt, Bitterfeld). Both resins are of angiosperms origin (flowering plants), and are classified into Class II based on Pyrolytic Gas Chromatography coupled with Mass Spectrometry (Py-GC–MS), and Group B based on Nuclear Magnetic Resonance Spectroscopy^36^. The characterization of the resins was carried out by means of optical observations, microhardness testing supported by various spectroscopic methods, such as: Fourier Transform Infra-Red Spectroscopy (FT-IR), Raman Spectroscopy (RS), Fluorescence Spectroscopy (FS), ^13^C Nuclear Magnetic Resonance (NMR), and Gas Chromatography-Mass Spectrometry (GC–MS). The aim of the study was also to find the most selective analytical tool for tracking the difference in chemical composition of fossil resins from a similar botanical origin that formed and altered in different geological conditions. Furthermore, this contribution is a kind of resumption of our previous studies on Sumatran resins^16^, providing additional data on their similarities and differences to glessite, which coexist within the same classification based on GC–MS class and NMR group.

## Geological settings and palaeogeographic background

### Sarolangun mine, Sumatra, Jambi Province, Indonesia

The investigated specimens of Indonesian resins come from the waste dumps of Sarolangun mine in the South part of the Sumatra Island. The fossilized resins were found within coal and lignite beds dating to the early Miocene (20–23 Ma). Stratigraphically, these deposits belong to Talang Akar Formation, the part of South Sumatra Basin^22^. The size of the resins varied, from very small (few mm in a diameter) to very large specimens (weighting several hundred grams).

**Figure 1:**
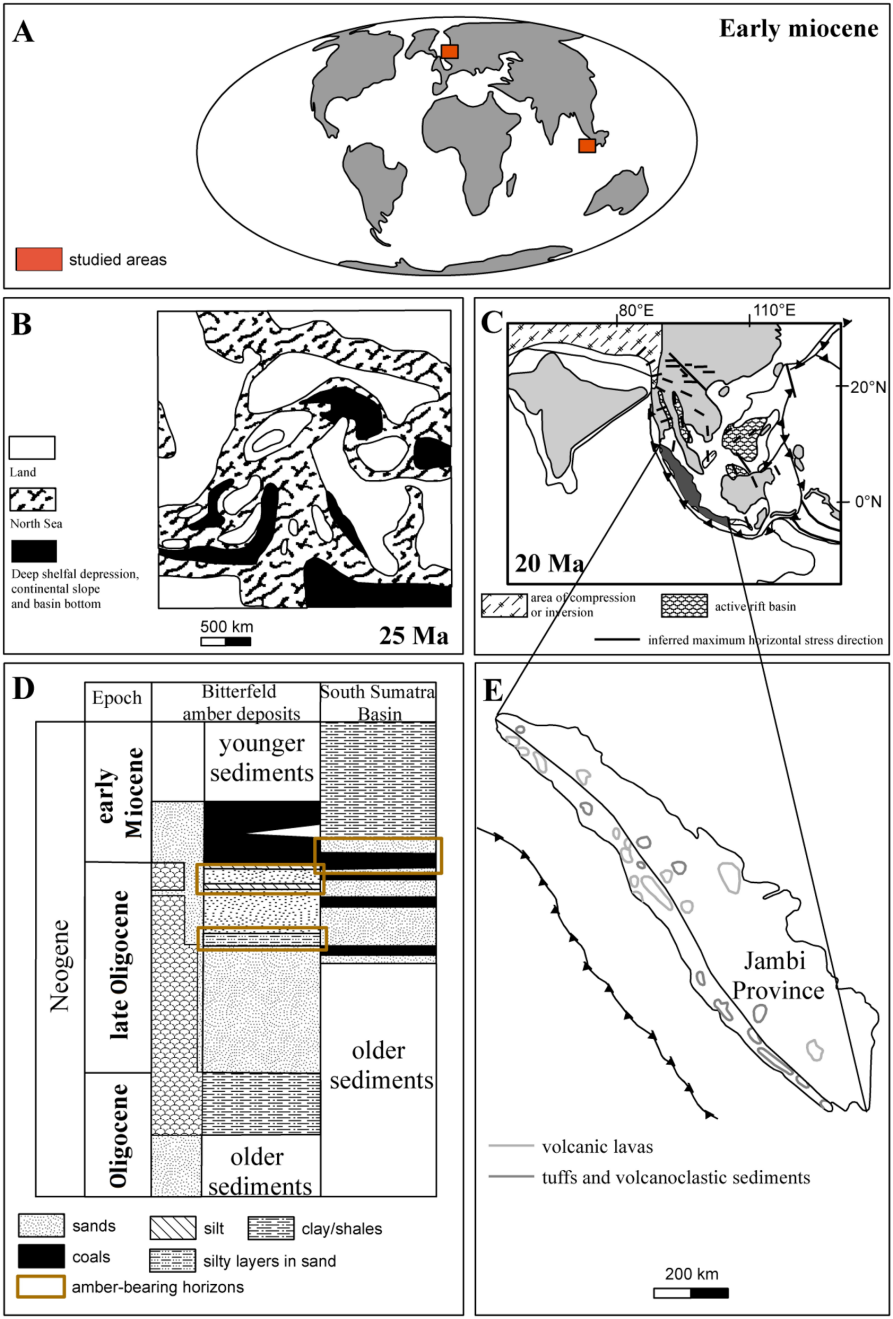
(**A**) Location of the Sumatra Island and Bitterfeld deposit (Germany) on the world map^16^; (**B**) The Bitterfeld region in the middle Miocene (25 Ma)^40^; (**C**) Tectonic map of South Asia including Sumatra Island (20 Ma)^16,39^; (**D**) The stratigraphy of South Sumatra Basin and Bitterfeld amber deposits^16,41,46^; (**E**) volcanic units on the map of the Sumatra^46^.

The Talang Akar Formation represents a sedimentary cycle extended from late Oligocene to the early Miocene epoch^37^. It is the retrogressive unit with shales and sandstones, deposited in a fluvio-deltaic environment building the upper level of the formation profile^38^. This unit is overlain by the early and middle Miocene sediments, representing the transgressive depositional systems. All of those formations were uplifted, folded and faulted due to the Pliocene–Pleistocene orogeny^37^, associated with volcanic activity (Fig. 1).

During the Cenozoic era, Sumatra had a warm and humid climate. It was a part of southern Sundaland located at latitudes ± 10° of the equator, with a palaeoclimate favorable for peat formation^39^.

### Germany, Saxony-Anhalt district, Bitterfeld deposit

The samples of glessite originate from the Bitterfeld region (Fig. 1) which is considered to be the largest deposit of brown coal in the district of Saxony-Anhalt, Germany^32,40–46^. The Bitterfeld fossil resins are linked to the Bitterfelder Bernsteinschluff horizons in the upper level of the late Oligocene Cottbus Formation, which consists of sediments of marine, fluvial and limnic origin^27,47–50^. The pieces of the resin are found in clay to sandy layers, associated with coal overgrowth and light dioctahedral mica. The resins-bearing sediments overlay the coal deposits^50^. The presence of marine plankton and glauconite suggests that these sediments were a result of ingress from the basin of north-west Europe^29^. Recently, age of the fossil resins-bearing sediments in Bitterfeld region was assigned to late Oligocene (Chattian; 23.0–28.1 Ma)^33,49^.

The palaeoenvironment of Europe during the Tertiary period was also influenced by warm and humid climate prevailing over the growth of forests, where conifers and other flowering plants, ferns and mosses coexist^50^. Moreover, large areas with the savanna-like character were also present in the environment. Some areas, like swamp forests, were frequently flooded with water. At that time, subtropical vegetation in the area of present Europe was more abundant than in the Sundaland, and had a larger territorial range due to the Eocene climate optimum.

## Results and discussion

The specimens of transparent variety of Sumatran resin, selected for detailed tests, are usually over a dozen mm in diameter (four samples). Specimens of German resins, glessite (tree samples), were also of similar size. Unlike the German specimens, the Sumatran resins show clear blue fluorescence under Schneider UV light, with 365 nm wavelength (Fig. 8).

**Figure 2:**
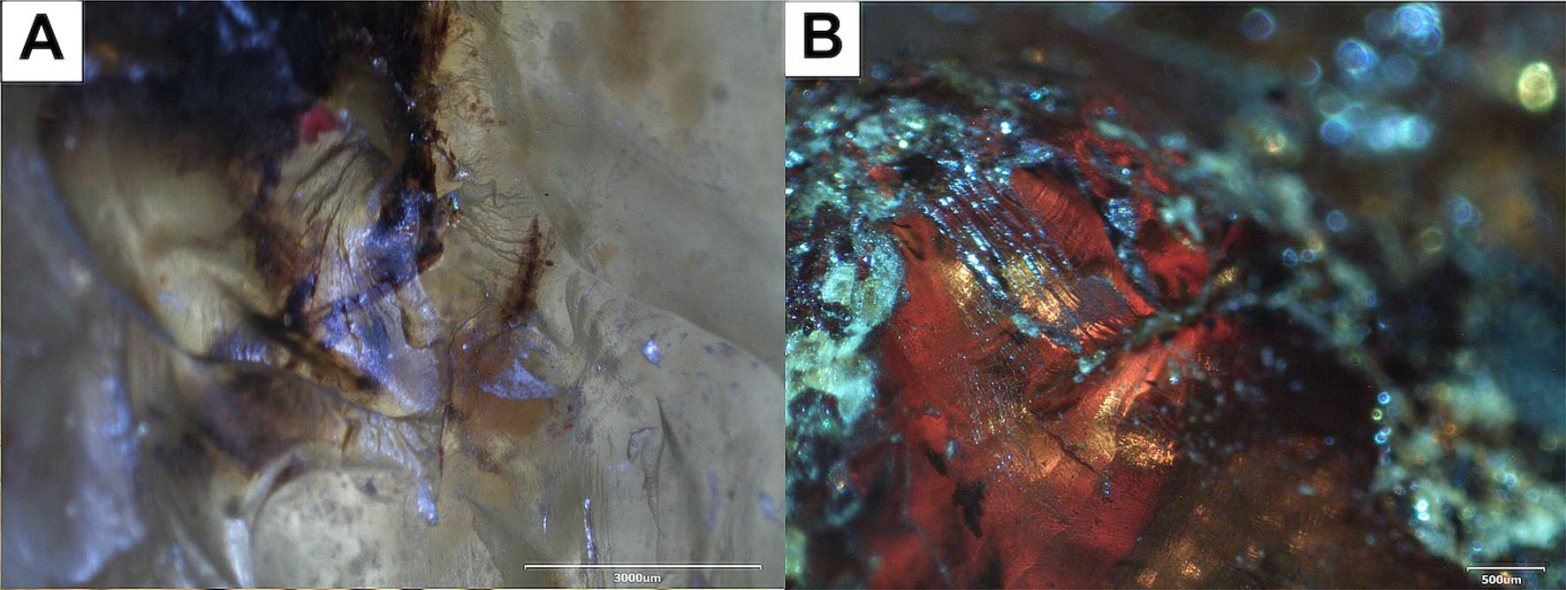
Images from a stereoscopic microscope; (**A**) numerous, mainly organic (dark) but also mineral and fluid inclusions in Sumatran resin; (**B**) the heterogeneous structure of Bitterfeld glessite.

Sumatran samples have distinctive visible characteristics under a stereoscopic microscope. The homogeneity of their yellowish colour was only disturbed by local streaks of congealed resin and low number of very small, organic inclusions, probably remains of wood tissue or bark as well as microspores of oval shapes (Fig. 2A). German resin samples are darker, brown–red and translucent. The number and varieties of inclusions in these resins seems to be greater (Fig. 2B).

The measured microhardness (H) of the Indonesian and German samples differed slightly. The H values (n = 30) varied for four samples of Sumatran resins within a narrow range: the minimum **H**_**vmin**_ = 19.62 (192.41 MPa) and the maximum **H**_**vmax**_ = 33.49 (328.42 MPa), the average (**H**_**va**_) value is 26.30 kgf/mm^2^ (257.91 MPa); **SD** = 3.66 kgf/mm^2^ (35.89 MPa). In the case of German specimens (three samples), microhardness values varied more significantly, from 14.12 to 40.33 kgf/mm^2^ (138.47–395.50 MPa), the average (**H**_**va**_) value was 27.44 kg/mm^2^ (269.09 MPa); **SD** = 7.98 kgf/mm^2^ (78.26 MPa). The results are summarized in Table 1.

**Table 1:** Microhardness of fossil resins from Sumatra and glessite from Bitterfeld.

Parameter	Fossil resin from Sumatra Island	Glessite from Bitterfeld
Arithmetic mean of microhardness Hav (kG/mm^2^)	26.30	27.44
Range of variability (kG/mm^2^)	19.62–33.49	14.12–40.33
Standard deviation	3.66	7.98
Standard deviation of arithmetic mean	0.819	1.78
Student’s t factor	2.09	2.09
Expanded uncertainty	1.71	3.73
Microhardness value x (kG/mm^2^)	26.29 ± 1.7	27.44 ± 3.73
Relative error ε± (%)	6.50	13.59

The development of spectroscopic methods constituted a breakthrough in the fossil resin studies, and have brought new possibilities in terms of their characterization and identification^4^. The FT-IR studies of Sumatran resins, previously performed by Kosmowska-Ceranowicz and Vávra^35^ and Matuszewska^9^, have shown that their spectra were very similar to the spectra of glessite from the Bitterfeld deposits. Any observable differences should reflect the transformation processes occurring during their diagenesis as they originate from similar biological angiosperm precursors^20,32^.

**Figure 3:**
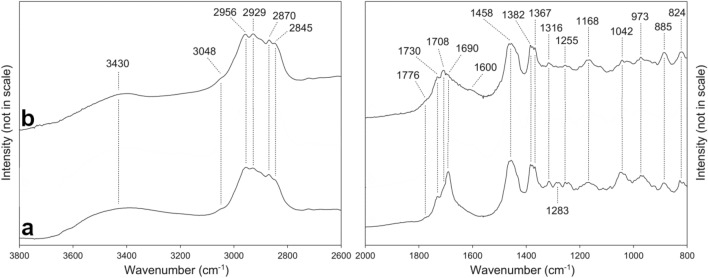
FT-IR spectra of Bitterfeld glessite (**a**) and Sumatran fossil resin (**b**).

The comparative results of our FT-IR studies of Sumatran and German resins (Fig. 3) confirmed those observations, however, some differences in the shape of the spectra in the region closed to ~ 1700 cm^−1^ were noticed (so-called *fingerprint area*). The Bitterfeld glessite (Fig. 3a) has the most symmetric C=O vibration band with a maximum of 1690 cm^−1^, while Sumatran resins show bands at 1776, 1730, 1708 and 1690 cm^−1^. Peak splitting of C=O band was observed on glessite spectra. This suggested that the surrounding environment of the carbonyl group is different in comparison to that of the analysed Sumatran specimens. The characteristic band for glessite occurs at ~ 1644 cm^−1^ while in the case of Sumatran resins, it is at a lower wavenumber of 1600 cm^−1^. The 1644 cm^−1^ band is characteristic of the exocyclic methylene group (vibration of double bond) while the band at ~ 1600 cm^−1^ could be assigned to the ester group^4^. Apart from those differences the spectra of both resin varieties are very similar within the range of 1350–800 cm^−1^, where additional bands occur, due to vibrations of the C–C and C-H bonds. Hence, these results confirmed that the most diagnostic IR bands occur in the 1800–1600 cm^−1^ region. The presence of the ester group on the Sumatran resins IR spectra suggests that polymerization (polyestrification) processes were involved in fossilization^10,16^. However, in the case of glessite, the resin was likely altered by the oxidation process. These findings could be well correlated with the geological background of studied resins. Glessite deposits were likely formed in a shallow-marine environment in the presence of dissolved oxygen.

Raman spectroscopic method (RS) is becoming widely used for geological purposes. It is sensitive and selective tool for the analysis of the organic materials. Over the last few years RS has also been adopted for studies of fossil resins mostly due to the simplicity of its use and non-destructive character^10,15,51^. Raman Spectroscopy is a method complementary to Infrared Spectroscopy as it enables identification of additional functional groups in IR spectra. Therefore, it is believed that RS could be the basis for further differentiation of fossil resins. Moreover, the shape of the Raman spectra of fossil resins is considered as an indicator of their maturation grade. Comparative Raman spectroscopic studies of differently aged fossil resins performed by Winkler et al.^8^ have shown, that their maturation processes are reflected in RS spectra details; nevertheless, some influences of thermal events during resin fossilization on these spectra shape were also observed. In the studied case, the intention was to identify Raman spectra similarities and/or differences between diversely aged fossil resins in relation to their formation and alteration environments.

**Figure 4:**
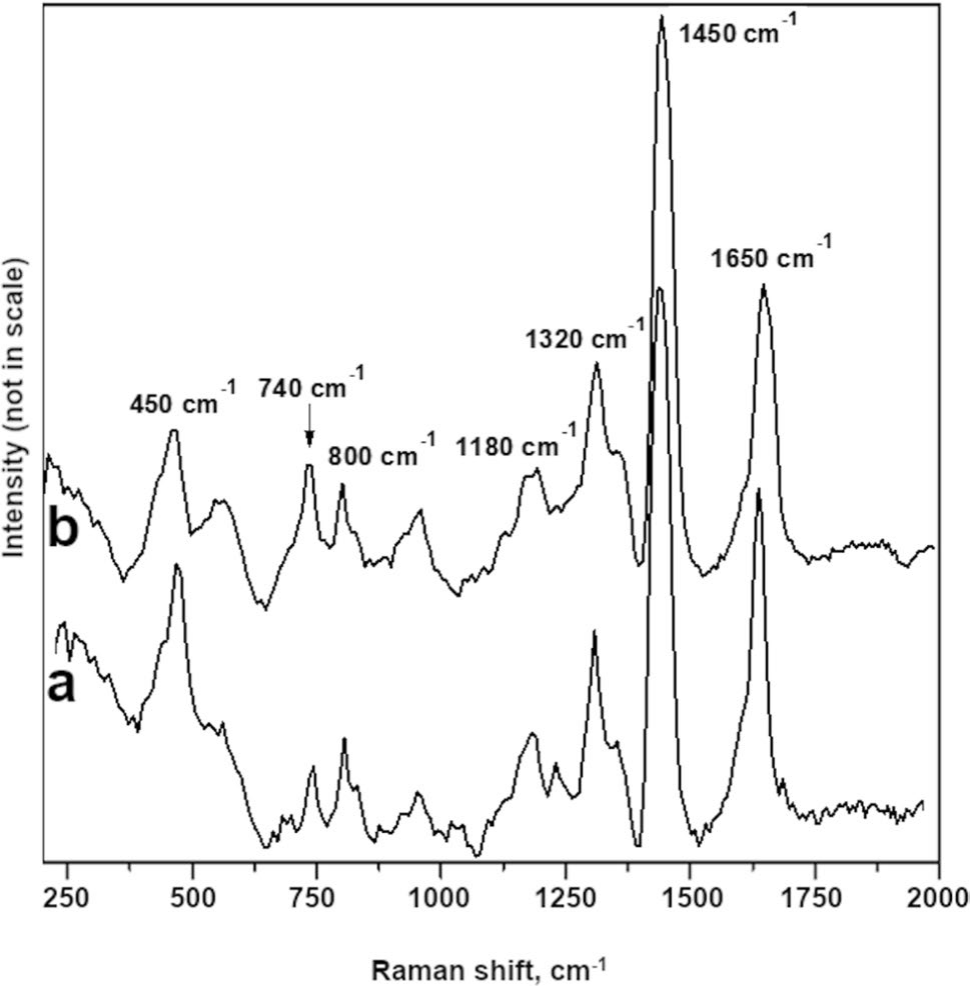
Raman spectra of Bitterfeld glessite (**a**) and Sumatran fossil resin (**b**) at a wavelength of 1064 nm.

Fossil resins from Sumatra Island produce similar Raman spectra to those of the glessite (Fig. 4). The RS spectra of all analysed samples were not obscured by the presence of water and hydroxyl groups and bands from symmetric vibrations C–C and C=C were also clearly visible. The most intensive and simultaneously most diagnostic bands for the interpretations of maturation processes of fossil resins are ~ 1640 cm^−1^ and ~ 1440 cm^−1^ bands. The 1640 cm^−1^ band is related to the stretching vibrations of C=CH_2_ (unsaturated hydrocarbons), while the 1440 cm^−1^ band is usually assigned to the deformation mode of the CH_2_ group or an asymmetric deformation of CH_3_ groups with unsaturated CH_2_ groups^8^. In the studied case, those diagnostic bands are shifted to the higher frequencies of ~ 1450 and ~ 1646 cm^−1^ (Fig. 4) respectively, but they are clearly distinguishable (the signals are at least three times higher than background). During diagenetic processes, cyclization and compound cross-linking lead to a decrease of double bonds. It may be assumed, that the first stage of diagenetic processes leads to the formation of unsaturated compounds (with one or more double bond) and further cross-linking/polymerization or cyclization^52^. Therefore, the ratio of intensity of a signal at 1646 cm^−1^ and 1450 cm^−1^ (I^1646^/I^1450^) may be interpreted as a degree of fossil resin maturation. In the studied case, the band of ~ 1450 cm^−1^ has a greater intensity than ~ 1646 cm^−1^ on both spectra. The intensity ratio of ~ 1646 cm^−1^/~ 1450 cm^−1^ is 0.56 for the resin from Sumatra and 0.68 for glessite.

Despite the younger age of Sumatran resins (Miocene), their I^1646^/I^1450^ intensity ratio was lower than that of Bitterfeld glessite, dated to the late Oligocene epoch. This may suggest that Sumatran resins underwent more advanced polymerization processes. However, it is worthwhile to mention that transformation processes are not only caused by age-dependent parameters, such as burial depth and geothermal gradient, but also by certain diastrophic events, i.e. development of magmatic intrusions or volcanic activity. The fossil resins from Sumatra are associated with layers containing tuffs^14^, therefore the volcanically-induced heating could explain why their I^1646^/I^1450^ ratio is lower than previously expected^10^. However, the presence of the opposite process to maturation also cannot be excluded. Oxidation may lead to formation of carbonyl and/or hydroxyl group, which may reverse the cross-linking process. Additionally, some microbiological oxidation of fossil resins in the deposition environment may affect the shape of their Raman spectra. The Sumatran resins are found in a coal layer, thus their depositional environment was rather reducing (dysoxic). In such conditions, processes leading to the formation of unsaturated compounds are prevalent.

Both of the Raman spectra show also relatively many details within the range of 2000 and 200 cm^−1^ (additional bands at 1320, 1180, 800 and 740 cm^−1^). These bands corresponding to the presence of CH_2_ and CH_3_ groups in saturated compounds are poorly resolved and visible on spectra. However, in the case of glessite, those bands are more distinguishable (Fig. 4b). It may indicate that Bitterfeld glessite is less matured than Sumatran resin. The presence of the band at 800 cm^−1^, corresponding to aromatic hydrocarbon deformations^53^, has been previously interpreted as a specific feature of Raman spectra of Indonesian resins^10^. However, this band has also been observed on Raman spectra of Bitterfeld resin. Thus, it could be attributed to the affinity of the resins rather than their diagenesis environment as was previously concluded^10^.

Fluorescence spectroscopy may provide new information on chemical composition of amber. The source of fluorescence is a large number of compounds embedded in fossil resins that are often responsible for their unique appearance^54^. The presence of fluorescent unsaturated and aromatic compounds in fossil resins from different worldwide localities are often reported in literature^15,19,32,54–60^. Fluorescent properties of those compounds are well known and reported in large number of papers^61^. Nevertheless, applicability of this method for study of fossil resin has not been validated in the literature so far^4^. The resin specimens are heterogeneous, anisotropic material, and thus the fluorescence excitation and emission spectra may vary from point to point. Therefore, finding any regularities within fluorescence emission-excitation spectra, and development of fluorescence-based method for identification of fossil resins requires numerous studies on a large number of specimens. In these investigations, fluorescence spectroscopy was used in order to reveal the presence of chemical constituents, which are the most likely responsible for Sumatran resin fluorescence that is observed under UV light, in contrast to non-fluorescing glessite.

**Figure 5:**
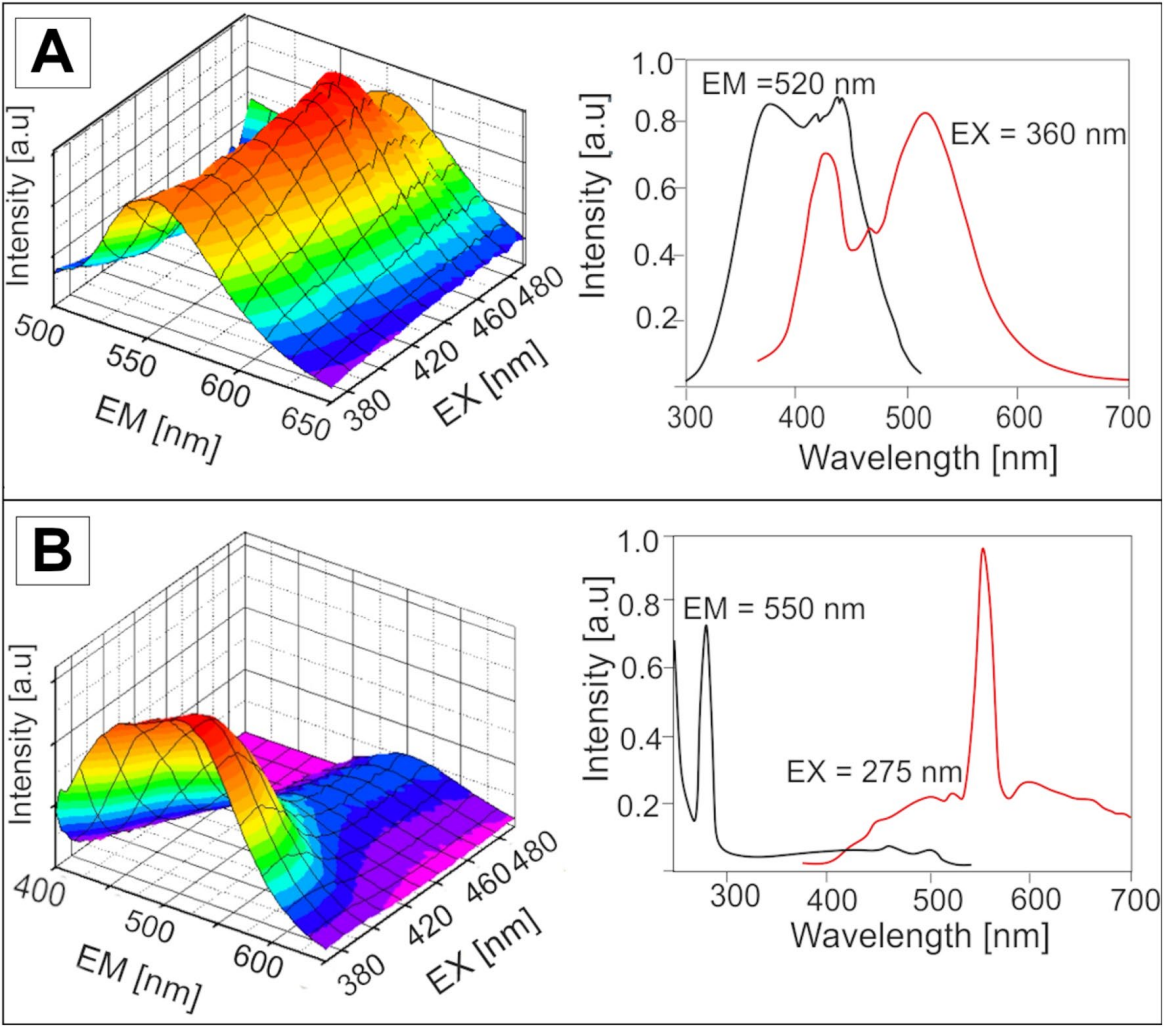
(**A**) Excitation–emission (EM–EX) spectra of Sumatran resin; (**B**) excitation–emission (EM–EX) spectra of Bitterfeld glessite.

Fluorescence spectra of the Sumatran resins (Fig. 5A) resemble the spectra of compounds with four or more aromatic rings^62,63^. Those compounds may form from cyclic terpenes that underwent an aromatization process under temperature and pressure conditions^64^. The presence of such constituents (i.e. perylene) was previously proposed as a source of blue fluorescence in resin observed under UV light^54,65^. However, in a later study, the presence of perylene in blue-fluorescing amber from Far East Russia was not confirmed^66^. Therefore, the interpretation of fluorescence spectra requires further chemical proof.

With regards to glessite, excitation-emission spectrum (Fig. 5B) is different than that of specimen from Sumatra. At the same measurement settings, the fluorescence intensity of glessite is around 20 times lower than that of Sumatran resin. It may be explained by the presence of other non-fluorescent compounds, absorbing the fluorescence light. However, the very strong and sharp emission band at 550 nm is likely a result of second order diffraction of light at 275 nm^61,67^. In the case of strongly fluorescing substances, this interference is not observable.

**Figure 6:**
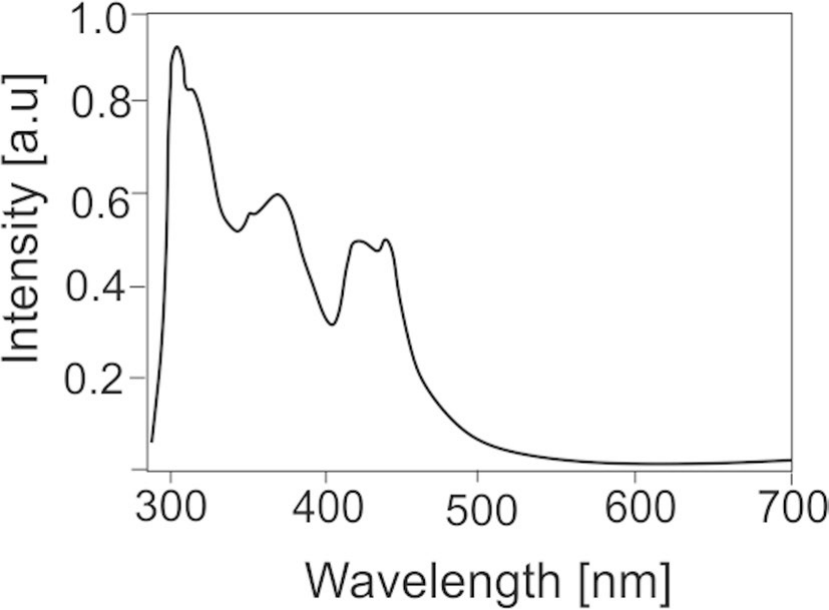
Synchronous fluorescence spectrum of Sumatran resin (λ_em_ = λ_ex_ + 18 nm).

Synchronous fluorescence was previously used for identification and determination of polycyclic aromatic carbon in Baltic amber^68^. However, in our study, synchronous fluorescence spectrum (λ_em_ = λ_ex_ + 18 nm) was only obtained for Sumatran resin (Fig. 6). The presence of polycyclic aromatic compounds in Sumatran resins is confirmed. Taking together the shape of synchronous fluorescence spectrum and excitation and emission spectrum with the excitation maximum at 360 nm and the emission maximum at 520 nm, it may be assumed that aromatic compounds with five and more rings were present in chemical structure of Sumatran resin^62,69^. It is still unknown whether such compounds are a part of polymeric lattice, or they are merely inclusions. This needs further investigation. The chemical structures of aromatic compounds hold information about sedimentary environment. The compounds may be formed during diagenetic transformation of biological molecules or they may form due to fire or heat effects. Moreover, different aromatic compounds are formed in lacustrine and marine environments. Therefore, the complex excitation-emission spectra needs also further studies by chemometric methods, in order to resolve spectra of fluorescence compounds present in the resin matrices. Fluorescence spectroscopy is a complementary method to infrared and Raman Spectroscopy. Taking together, the results obtained by the use of these methods enable researchers to reveal some information about the chemical structure of the resin without destruction of the specimen.

**Figure 7:**
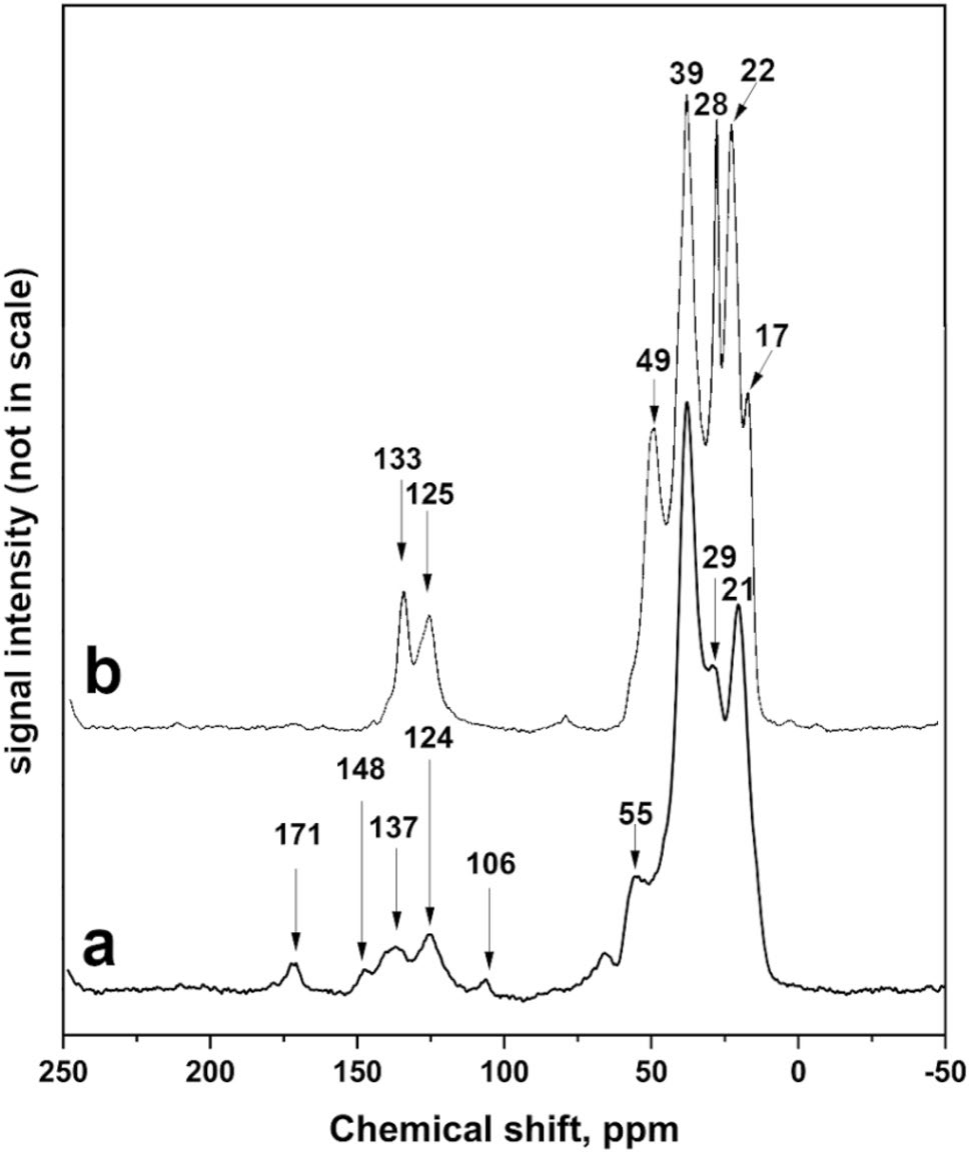
^13^C NMR spectra of Bitterfeld glessite (**a**) and Sumatran fossil resin (**b**).

Recently, application of ^13^C solid state Nuclear Magnetic Resonance (NMR) spectroscopy in study of fossil resin has been demonstrated to be a powerful method for obtaining botanical and geological information^70^. Examination of the solid-state ^13^C NMR spectra of fossilized resins (ambers) has generated five groupings of materials based on spectral characteristics related to carbon functionalities^70^. According to this classification, resins from Sumatra and glessite from Bitterfeld belong to the same NMR Group B, the group of resin originating from angiosperms or their precursors (flowering plants)^36,71^. In the spectra of both resins, the most intense resonances were found in the saturated region, δ 0–60 (Fig. 7). These are from saturated carbons typical of terpenoid hydrocarbons^70^. Contrary to Bitterfeld resin, in spectra of Sumatran resin, there were no weaker resonances in the region in which saturated carbon that is substituted with electron-withdrawing groups such as oxygen functionalities (δ 60–110) resonates. The strong differences were observed in unsaturated region δ 100–160, which contains resonances from carbons of double bonds. Singly substituted alkene carbons (C–HC=CH–C) resonate in the range δ 120–145, unsubstituted alkene carbons (C=CH_2_) at δ ca. 110, and disubstituted alkene carbons (> C=C) at δ ca. 150. In spectra of Indonesian resin, narrow and sharp peaks corresponding to resonances at δ 133 and 125 suggest the presence of aromatic compounds. In the case of Bitterfeld resin, the resonances at both δ ca. 110 and ca. 150 strongly suggest the presence of an exomethylene or terminal group (> C=CH_2_)^70^. Peak at δ ca 170 suggests presence of carboxylic group in structure of German resin. This peak is observed also in spectrum of Baltic amber and corresponds to presence of succinic acid.

Despite the numerous investigations that have been conducted, the chemical composition of various fossil resins needs further elucidations, especially in terms of their diagenetic alterations from plant exudate to their present fossil form. Analytical methods based on mass spectrometry were successfully applied in the fossil resin studies of many authors^17,32,56,58,59,72^. Application of mass spectrometry gives in-depth information about chemical structure of the fossil resins. According to Anderson et al.^17^, the chemical profile of resins compounds can be used for their classification. Additionally, chemical analysis provides information on botanical origin of studied resins and their evolutionary pathways after deposition. The method used commonly for classification and chemical analysis of resins is Py-GC–MS. However, this method is used mainly for analysis of polymerized resin materials^17^. Some resin constituents may not be polymerized; the compounds may be embedded into polymer lattice. Therefore, solvent extraction combined with gas chromatography is frequently used for analysis of those compounds^32^.

The chemical composition of both Sumatran and German resin samples contains predominantly sesquiterpenoids and triterpenoids (Table 2). The chemical profile of triterpenoids was similar in resin specimens and consisted of betuline, alfa-amyrin and lupeol (hydroxylupene). The chemical analysis of Sumatran resin confirmed that botanical source was Dipterocarpaceae family of SE-Asian trees^20^. The presence of betuline and luepol is usually considered as characteristic of angiosperms^73^. Both resins contained similar profile of triterpenoids with the oleanane, ursane and lupane structures. This profile indicates botanical source as angiosperm^32,74–76^. The alfa-amyrine, non-altered product of higher plants, and characteristic component of both resins is typical biomarker of an angiosperm plant^9,30^. This component is also believed to originate from Burseraceae—a family of flowering plants found on both areas of present European and Asian continents during the Cenozoic era^73^. The similar botanical affinities of resins may be explained by the fact that during the late Oligocene—Miocene epoch climate was warm and humid contrary to present climates in present areas of Europe and Asia^32,77,78^. Biomarkers, such as aromadendrene-type sesquiterpenoid were only present in solvent extract of Sumatran resin. These components have been previously documented in *eucalyptus* oils^79^. On the other hand, allobetuline found in the glessite from Bitterfeld indicates birch (*Betula*) as the possible resin producing tree^32^.

**Table 2:** Summary of chemical analysis of fossil resins from Sumatra Island and glessite from Bitterfeld.

Fossil resins from Sumatra Island			Glessite from Bitterfeld deposits (Yamamoto et al.^32^)		
Compound	MW	Composition	Compound	MW	Composition
**Aromatic hydrocarbons**					
Toluene	92	C_7_H_8_	1,4-Dimethyl-1,2,3,4-tetrahydro-naphthalene	160	C_12_H_16_
1,3-Diethyladamantane	192	C_14_H_22_			
**Sesquiterpenoids**					
**Cadina-1(10),6,8-triene**	202	C_15_H_22_	**Cadina-1,6-diene**	204	C_15_H_24_
**Cadina-1,3,5-triene (l-calamenene)**	202	C_15_H_22_	**Calamenene**	202	C_15_H_22_
**β-Cadinene**	204	C_15_H_24_	**Calamene**	202	C_15_H_22_
**α-Muurolene**	204	C_15_H_24_	**Cadalane**	198	C_15_H_18_
*Aromadendrene*	204	C_15_H_24_	**Dihydro-ar-curcumene**	204	C_15_H_24_
			**β-Caryophyllan-2,6-oxide**	222	C_15_H_26_O
**Triterpenoids**					
*Betuline*	442	C_30_H_50_O_2_	*28-Nor-β-amyrin isomer*	412	C_29_H_48_O
*α-Amyrin*	426	C_30_H_50_O	*28-Nor-α-amyrin isomer*	412	C_29_H_48_O
*Lupeol*	426	C_30_H_50_O	*28-Nor-β-amyrone*	410	C_29_H_46_O
*Urs-12-ene*	410	C_30_H_50_	*28-Nor-α-amyrone*	410	C_29_H_46_O
			*28-Nor-β-amyrin*	412	C_29_H_48_O
			*β-Amyrin*	426	C_30_H_50_O
			*28-Nor-α-amyrin*	412	C_29_H_48_O
			*α-Amyrin*	426	C_30_H_50_O
			*Allobetul-2-ene*	424	C_30_H_48_O
			*3β-Lupeol*	426	C_30_H_50_O
			*Erythrodiol*	442	C_30_H_50_O_2_
			*Lupeol isomer*	426	C_30_H_50_O
			*Uvaol*	442	C_30_H_50_O_2_
			*Oleanolic acid*	456	C_30_H_48_O_3_
			*Ursolic acid*	456	C_30_H_48_O_3_
			*Oxyallobetulane*	440	C_30_H_48_O_2_
			*Allobetulin*	442	C_30_H_5_0O_2_
			*Ursa-(1/2/5/6),12-dienolic acid*	452	C_30_H_44_O_3_

*MW* molecular weight.

Bold—biomarkers indicating diagenetic pathways of resins, italics—biomarkers indicating biological origin of resins.

Chemical composition of fossil resins provided also a source of information about their fossilization history. Cadinene-type compounds from sesquiterpenoids group undergo diagenesis to cadalene derivatives^80^. Moreover, sesquiterpenoids have shown the greater susceptibility to diagenetic alteration than pentacyclic triterpenoids^81^. Compounds with a cadinene moiety were found in German and Indonesian resin specimens. In the case of fossil resin from Sumatra Island, the presence of intermediate product of diagenesis—Cadina-1(10),6,8-triene, indicates the relatively advance stage of its maturation^80^. This corroborates the results of Raman Spectroscopy studies. In the Bitterfeld glessite, the presence of cadalane, the final product of diagenetic alteration, suggests more advanced maturation; however, this is not consistent with results of Raman Spectroscopy studies, which indicate less mature resin. This may be explained by the fact that the processes of sesquiterpenoids transformation are affected by the inorganic environment^80^. The glessite occurs in glauconite-rich layers; therefore, the deposition environment is chemically diversified, in some cases, rich in iron. The presence of iron may affect the diagenetic transformations of sesquiterpenoids biomarkers^80^.

In applied analytical conditions, aromatic compounds were not found in Sumatran resin. Source of toluene in resin is likely contamination after excavation and transportation to the laboratory. To explain the presence of polyaromatic compounds in the investigated resin samples, product of diagenesis of amyrin, botulin and lupeol, cannot be excluded^64^.

In conclusion, the fossil resins of a similar botanical origin but a different preservational history were investigated. Both resins from Sumatra Island (Jambi Province, Sarolangun mine) and Germany (Saxony-Anhalt, Bitterfeld deposits) originate from exudates of trees belonging to angiosperms species. However, they were formed in various palaeoenvironmental conditions and deposited in different sedimentological systems, thus they were subjected to different alteration processes from the time of their formation to the present form. Factors which differentiate between these palaeogeographical and geological environments are: solar light, redox conditions, and thermal maturation of resin-bearing sediments (not strictly related to their age). In general, fossil resins from Sumatra Island and Germany were formed under similar palaeogeographical conditions (similar biocenosis of amber-bearing forests, warm and humid climate), but the latitude of their occurrence differed, which corresponds to the various solar light intensity. Therefore, this may influence the resins’ chemical signature at the time of their formation. Additionally, in the case of Sumatran resins, dysoxic conditions and volcanic activity likely occurred during their burial. Subsequently, the resins were subjected to various diagenetic alterations when further differentiation was possible due to the different sedimentological and diagenetic systems. Having the knowledge about transformation pathways of organic compounds from burial to present time, one can determine original chemical content of plant exudate. The chemical content is further used in chemotaxonomy of plant species.

In this study, the results obtained with the set of analytical methods confirmed that Sumatran fossil resins show significant similarity to the glessite from Bitterfeld. Both resins have similar spectroscopic characteristics, as IR and RS spectra recorded for them are nearly identical. The most important feature, which differentiates resins, is chemical composition, especially the presence of some components responsible for producing their fluorescence spectra (aromatic hydrocarbons). Hence, Gas Chromatography–Mass Spectrometry and Fluorescence Spectroscopy were the most sensitive tools to identify any variations of chemistry between Sumatran and German resins. Nuclear Magnetic Resonance spectroscopy permits additional differentiation of resins, which allows to track changes in spectra according to resin maturity and diagenetic history.

The degree of compound aromatization may be used for assessment of the resin maturation. This can be easily indicated by Fluorescence Spectroscopy, without destruction of the resins. The results of ^13^C NMR study indicated the presence of carboxylic acids, oxidation products of terpenoids, in Bitterfeld resins. The mechanism of carboxylic acid formation in fossil resin is still disputable.

## Material and methods

### Material

**Figure 8:**
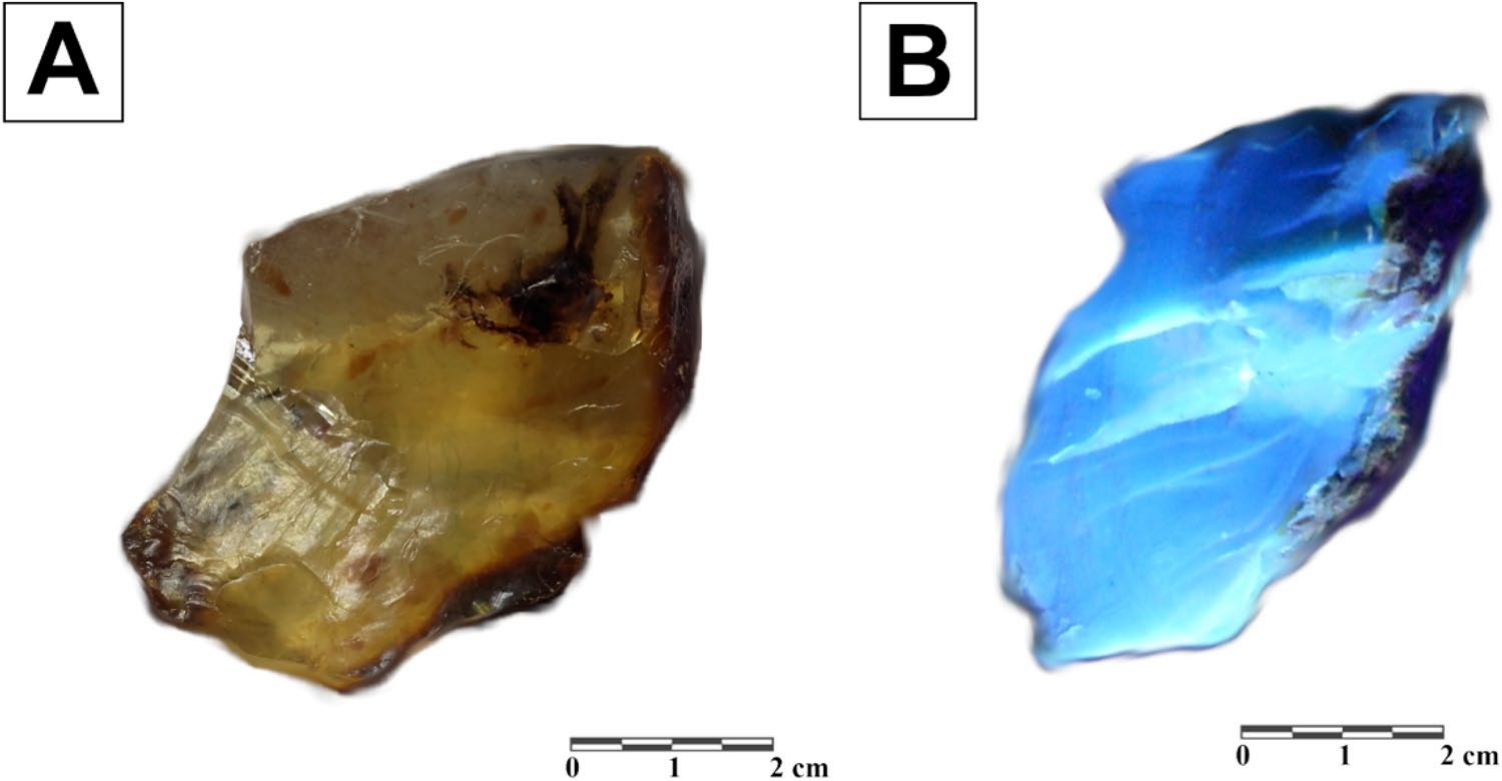
Specimen of Sumatran fossil resin under a white light (**A**) and UV light—365 nm (**B**).

**Figure 9:**
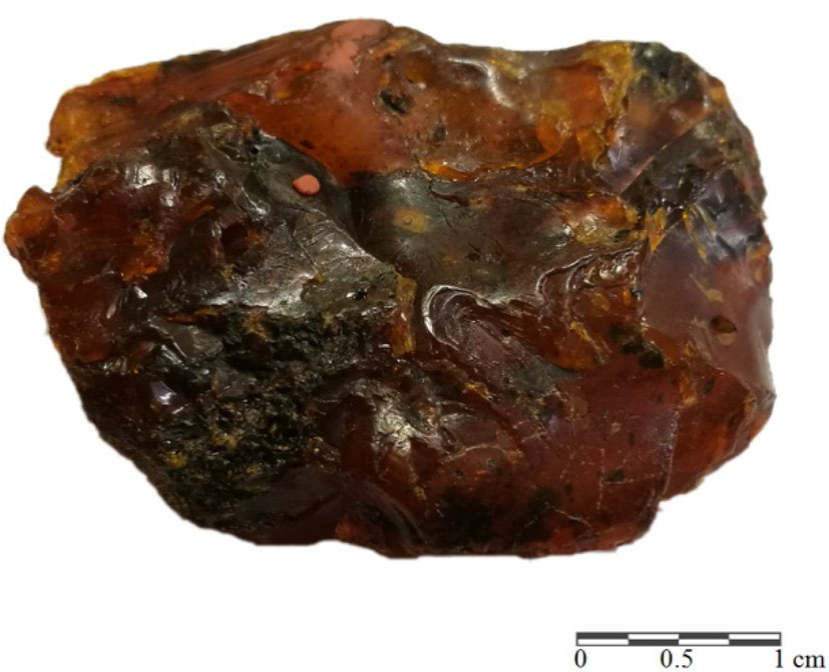
A Bitterfeld glessite sample.

For this study, the samples of resins from Sumatra (Fig. 8) were provided by Dr. Robert Girulski (University of Wroclaw, Poland). The specimens of transparent resin with light yellow colouration (I type described by Naglik et al.^16^), are considered as the less altered, and were taken for further analytical tests. Glessite samples from Bitterfeld (Fig. 9) came from Prof. Günter Krumbiegel’s collection housed in Halle (Germany) and were provided by his son, Dr. Anselm Krumbiegel.

### Stereoscopic microscope

Internal structures of resins and inclusions occurring in them were observed with the Motic SNZ-168 stereoscopic microscope, coupled with a Lusis digital camera (Model CMOS HC-30MU) and Huwitz Panasis software for macrophoto documentation. The research was performed in a laboratory of the Faculty of Geology, Geophysics and Environmental Protection of AGH UST, Kraków, Poland.

### Microhardness testing

Determinations of microhardness parameters were performed using a Russian made tester PMT-3 in the Gemological Laboratory of the Faculty of Geology, Geophysics and Environmental Protection of AGH UST, Kraków, Poland. The testing procedure was performed according to the Vickers method as described elsewhere^7^. It involves pressing a quadrangular diamond pyramid with an dihedral angle equal to 136° on the flat surface of the tested material. The tests were performed on the smooth surfaces of each of the four samples of resins from Sumatra and three samples of glessite, and the measurements were repeated 30 times. Subsequently, the results obtained were statistically processed.

### Fourier Transform Infrared Spectroscopy (FT-IR)

IR spectra of analytical resin samples were collected in a laboratory the Faculty of Geology, Geophysics and Environmental Protection of AGH UST, Kraków, Poland, using a Nicolet 6700 spectrometer (Thermo Scientific, USA) with 64 scans at 4 cm^−1^ resolution in the 4000–400 cm^−1^ mid-region. A mixture of 3 wt% sample/KBr was analysed by diffuse reflectance infrared Fourier transform spectroscopy (DRIFT) using a Praying Mantis Diffuse Reflection Accessory (Harrick). The DRIFT technique applied to the samples gave a higher signal to noise ratio compared with methods based on preparation of KBr pellet.

### Raman Spectroscopy

The Raman spectra of resins were recorded using the handheld Near-Infrared Raman spectrometer Progeny ResQ (Rigaku, Japan), equipped in spectrometer with InGaAs detector was loan from Tusnovics Instruments Ltd., Kraków (Poland). The spectrometer equipped with 1064 nm Nd:YAG laser line excitation, with a maximum power value of 490 mW. The laser line at 1064 nm was chosen in order to avoid strong fluorescence background^10^. The spectra were recorded in the range from 2000 to 200 cm^−1^ with 8 cm^−1^ resolution.

### Fluorescence Spectroscopy (FS)

Fluorescence emission and excitation spectra were measured with an LS900 spectrofluorometer from Edinburgh Instr. in the Institute of Optoelectronic in the Military University of Technology, Warsaw, Poland. The source of the excitation was a 450 W xenon lamp. The spectra were recorded at a resolution of 2 nm. Flat polished plates were cut from the specimens and ground. Fluorescence spectra were measured from the surface of the samples to avoid the internal filter effects. Emission–excitation spectra (EX–EM) were obtained with 5 nm resolution. Synchronous fluorescence spectra was obtained through the simultaneous scanning of the excitation and the emission with a fixed 18 nm wavelength difference between them.

### Nuclear Magnetic Resonance (^13^C CP-MAS NMR)

NMR analyses were made in the Nuclear Magnetic Resonance Laboratory at Łukasiewicz Research Network—PORT Polish Center for Technology Development, Wrocław (Poland). The measurements were carried out on a 14.1T Bruker III Advanced HD spectrometer operating at the ^13^C frequency of 150.13 MHz, equipped with a broadband 3.2 mm Cross Polarization Magic Angle Spinning (CP MAS) probe. Powder samples of 100–200 mg were packed into a 3.2 mm zirconia rotor. The spin rate was 20 kHz. Pulse power was 83 W and pulse width was 4 μs. The optimized contact time for cross-polarization was 2 ms. 4096 points were collected for one scan. The sample measurement was the sum of 8500 scans. Glycine was applied as a reference for the chemical shift (176.0 ppm).

### Gas Chromatography–Mass Spectrometry (GC–MS)

GC–MS analyses were made in a laboratory of the Earth Science Institute, the Slovak Academy of Sciences in Banská Bystrica (Slovakia). Small amount of Sumatran resin (20–50 mg) was pulverized in an agate mortar and transported into 2 ml vials. Then, a mixture of the organic solvents dichloromethane and methanol (9:1) was added to the vial and left for three days at 50 °C. Thereafter, samples were sonicated and filtered on Pasteur pipets filled with silica wool and connected to a glass manifold with a vacuum. Extracts were analysed by GC–MS (TraceGC Ultra—ITQ900, Thermo Fisher Scientific), on 60 m nonpolar capillary column Zebron ZB5, connected to an ion-trap quadrupole with an electron-impact ionization for collection of mass spectra. Helium was used as a carrier gas. The temperature program was the following: 60 min at initial temperature of 70 °C, with one minute holding, than ramped to 180 °C at 30 °C min^−1^ rate, and after that, ramped to 330 °C at 4 °C min^−1^ rate. Finally, temperature was kept at 330 °C for 16 min. Analysed data were processed with the Xcalibur software with mass spectra library (NIST library), obtained mass spectra were additionally analysed by comparison to mass spectra reported in literature. The GC–MS results obtained for resin from Sumatra were discussed alongside GC–MS glessite data published by Yamamoto et al.^32^.
